# Modeling the transmission dynamics of racism propagation with community resilience

**DOI:** 10.1186/s40649-021-00102-2

**Published:** 2021-11-06

**Authors:** Dejen Ketema Mamo

**Affiliations:** Department of Mathematics, College of Natural and Computational Sciences, Debre Berhan University, 445, Debre Berhan, Ethiopia

**Keywords:** Community resilience, Racism spread, *SERDC* model, Mathematical analysis, Numerical results

## Abstract

Racism spreading can have a vital influence on people’s lives, declining adherence, pretending political views, and recruiters’ socio-economical crisis. Besides, Web 2.0 technologies have democratized the creation and propagation of racist information, which facilitated the rapid spreading of racist messages. In this research work, the impact of community resilience on the spread dynamics of racism was assessed. To investigate the effect of resilience-building, new *SERDC* mathematical model was formulated and analyzed. The racism spread is under control where $R_0<1$ , whereas persist in the community whenever $R_0>1$. Sensitivity analysis of the parameters value of the model are conducted. The rising of transmission and racial extremeness rate provides the prevalence of racism spread. Effective community resilience decline the damages, mitigate, and eradicate racism propagation. Theoretical analysis of the model are backed up by numerical results. Despite the evidence of numerical simulations, reducing the transmission and racial extremeness rate by improving social bonds and solidarity through community resilience could control the spread of racism.

## Introduction

The propagation of racism in compound multicultural societies significantly affected all aspects of their life. The widespread proliferation of racism can lead to a series of serious hazards, such as social instability, impacts on election results, or large financial losses [15]. Racism can be viewed as mind infectiousness. The spreading and its effect indicted similar to epidemic diseases, like Coronavirus and Ebola virus. What is worse, racist activists are more likely to exist in societies and accepted as racially demarcated. This bad consideration has been propagating racism more quickly and widely [4].

The resilience theory has become increasingly essential not only to physical threats, but also to social challenges. The community faces social challenges from forces that threaten social cohesion and break down social assets. Racism can be identified as a force that separates and isolate minority groups, disengagement them from multiple social agendas. Besides, it reduces their influence on the development of the broader social aspect. Moreover, racism also fragments the groups themselves, allowing individuals quarantined from their peers. Further likely to act in destructive ways either against themselves or against those they see as their enemies. Racism provides public health consequences, and it can be considered a public health crisis [7, 22]. Resilience concerns the strength of a system to sustain itself through reform via friendship and casual transformation [1, 23].

Community resilience is the continued ability of a community to utilize available resources to respond to, confront, and recover from adverse situations. Moreover, a resilient community would decide what it requires to conquer damage and then sustain to strengthen health, social and economic systems. The community would learn from the past and enhance mobility, primarily through adequate social wealth advancement [8, 9]. It requires community resilience building and the brace of identity within convinced narratives and shared responses to threat. A resilience program identifies public affairs, needs systematic and transparent modes of solidarity. Community resilience admits communities to sense granted [3, 14]. Resilient communities, consequently, acquire to struggle beside, accommodate, and shape change, and such community resilience is a symbol of social sustainability [2, 19, 24].

Mathematical modeling has a vital role in system dynamics understanding of real-world phenomena in different aspects. It has been widely applicable in physical, biological as well as social sciences; see reference [5, 6, 10, 13, 16, 17, 20]. An effective mathematical model for spread control of racism will have principal theoretical significance in inhibiting the spreading processes, and then offers practical measures for minimizing losses induced by racism. The study of its spreading follows either model of opinion dynamics or a model of biological epidemics. Recently, [21] studied racism spreading in cyberspace and proposed the *SEID* model, in which population was separated into four categories: Susceptible (people who are unaware of the racism), Exposed (people who are aware of the racism, yet not decided to be racist or denier), Infected (people who spread the racism), Denier (people who are aware of the racism, but choose not to spread it).

This study is focusing on the assessment of community resilience’s effect on the spread of racism. The new model incorporates community resilience as a control strategy to minimize the harm induced by racism. The theoretical analysis, sensitivity analysis, and numerical simulation will be conducted.

## Model formulation

In this section, a racism spreading model, which considers the impact of community resilience (anti-racism) campaign, is proposed and referred as $$SERDC$$ model. Based on the classical SIR model and hesitating mechanism, the total population is divided into four classes: namely $$S(t)$$-susceptible population, $$E(t)$$-exposed population, $$R(t)$$-racist population, and $$D(t)$$-denier population. The class *S*(*t*) stands for people who do not know the racism. The class *E*(*t*) represents people who are aware of the racism, but hesitate to be racist and do not spread it. The class *R*(*t*) represents people who are racially racist and actively spreading racism. The class *D*(*t*) represents people who are aware of the racism, but choose to deny to spread the racism; individuals who have either stop spreading it or never spread it due to community resilience or anti-racism campaign. Furthermore, let *C*(*t*) be the cumulative number of community resilience building campaigns in the region at time *t*. Each individual must belong to only one of the four classes. Let $$\varLambda $$ be recruitment rate of individuals in the susceptible class. Racism increases through the contact of susceptible individuals with racial racists. When an individual in class $$S$$ comes into contact with an individual in class $$R$$ get to aware the racism, he or she will switch to $$ E$$ with a probability $$\beta $$, where $$\beta $$ is the spreading probability, which depicts the probability that a susceptible individual knows the racism via contact with an individual in class $$R$$. If the individuals are well-informed about the threat of racism, then they may have abstained from racist group propagandas. An anti-racism campaign mitigates the propagation of racism by creating awareness about racism threats. We consider the growth rate of the community resilience building is proportional to the number of racist individuals. The constant $$C_0$$ is the baseline number of community resilience, which is regularly operating in the community. The resilience campaign is targeted to induce behavioral change in susceptible individuals. Some portions of susceptible class individuals will become racism denier class by the rate $$\theta $$, where $$\theta $$ is the rate of effective community resilience campaign on susceptible individuals ($$\beta +\theta \le 1$$). Individuals in class $$E$$ can switch to class $$R$$ or $$D$$ depending on trust and interest about racism. We assume that individuals in class $$E$$ switch to class $$R$$ with the probability of $$\phi _r$$ when they decide to spread the racism and switch to class $$D$$ with the probability $$\phi _d$$ when they choose to deny racism spreading ($$\phi _r+\phi _d\le 1$$). Racially racist individuals convert to be a racist-denier class due to various reasons with the probability of $$\gamma $$ per unit time. Further, it assumed that some resilience community building campaigns fade or lose their impact on people. Moreover, the parameters $$\alpha $$ and $$\mu _0$$ are constants denoting the rate of implementation coverage and depletion of community resilience campaigns, respectively. The constant $$\mu $$ denotes the natural death rate.

**Fig. 1:**
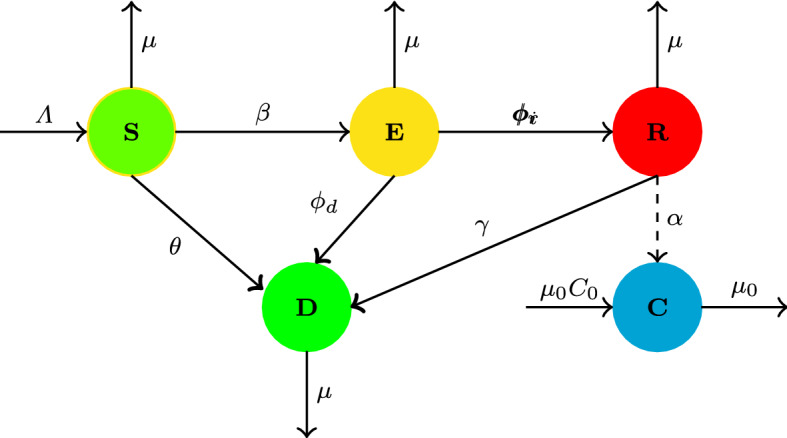
The flow diagram of the model

By considering the above assumptions and the schematic diagram in Fig. 1, the spread dynamics of racism with community resilience is described by using the following system of differential equations:

1 $$\begin{aligned} \begin{aligned} \dfrac{\text {d}S}{\text {d}t}&= \varLambda - \beta SR - \theta SC-\mu S,\\ \dfrac{\text {d}E}{\text {d}t}&= \beta SR- (\phi _d+\phi _r+\mu ) E ,\\ \dfrac{\text {d}R}{\text {d}t}&= \phi _r E- (\gamma +\mu ) R ,\\ \dfrac{\text {d}D}{\text {d}t}&= \theta SC+ \gamma R+\phi _d E -\mu D,\\ \dfrac{\text {d}C}{\text {d}t}&=\alpha R-\mu _0 (C-C_0), \end{aligned} \end{aligned}$$

where $$ S(0)>0, \,E(0)\ge 0 ,\,R(0)\ge 0 ,\, D(0)\ge 0 $$, and $$C(0)\ge 0$$.

The total population *N*(*t*) would be a constant all the time in this model, i.e.,

$$\begin{aligned} N=S+E+R+D=1. \end{aligned}$$

## Mathematical analysis

### Well-posedness

#### Theorem 1

If $S(0)\ge 0,\, E(0)\ge 0,\, R(0)\ge 0,\, D(0)\ge 0,\,$ and $C(0)\ge 0$, then the solutions $S,\, E,\, R,\, D,\,$ and $C$ of system (1) are positive for all $t\ge 0$.

#### Proof

From the first equation of the system (1), we obtain

2 $$\begin{aligned} \begin{aligned} \dfrac{\text {d}S(t)}{\text {d}t}&= \varLambda - \beta S(t)R(t) - \theta S(t)C(t)-\mu S(t) \ge - \beta S(t)R(t) - \theta S(t)C(t)-\mu S(t) \\ \dfrac{\text {d}S(t)}{\text {d}t}&+H(t)S(t)\ge 0 . \end{aligned} \end{aligned}$$

Let $H(t)=\left( \beta R(t)+\theta C(t)+\mu \right)$ and multiply both sides the last inequality of (2) by $\displaystyle {\text {exp}}{\left( \int _0^tH(s)\,{\text {d}}s\right) }$. We get

$$\begin{aligned} {\text {exp}}{\left( \int _0^tH(s)\,{\text {d}}s\right) }\dfrac{\text {d}S(t)}{\text {d}t}+\displaystyle {\text {exp}}{\left( \int _0^tH(s)\,{\text {d}}s\right) }H(t)S\ge 0. \end{aligned}$$

Then

$$\begin{aligned} \frac{\text {d}}{\text {d}t}\left( S(t) {\text {exp}}{\left( \int _0^tH(s)\,{\text {d}}s\right) }\right) \ge 0. \end{aligned}$$

Integrating this inequality from $0$ to $t$ gives

$$\begin{aligned} \int _0^t \frac{\text {d}}{\text {d}S(t)}\left( S(t) \displaystyle {\text {exp}}{\left( \int _0^tH(s)\,{\text {d}}s\right) }\right) \ge 0. \end{aligned}$$

Then

$$\begin{aligned} S(t)&\ge S(0) {\text {exp}}{\left( \int _0^tH(s)\,{\text {d}}s\right) }\\ S(t)&\ge 0. \end{aligned}$$

It can be shown in a similar way that $E\ge 0,\, R\ge 0,\, D\ge 0$ and $C\ge 0$. Therefore, all the solution sets are positive for $t\ge 0$. $\square$

Here note that

$$\begin{aligned} N(t)=S(t)+E(t)+R(t)+D(t). \end{aligned}$$

By combining the first four equations of model (1), we have

3 $$\begin{aligned} \frac{\text {d}N(t)}{\text {d}t}=\varLambda -\mu N(t)-\alpha R(t). \end{aligned}$$

So, the feasible domain of the system (1) is

$$\begin{aligned} \varGamma = \left\{ (S(t), E(t), R(t), D(t), C(t)) \in {\mathbb {R}}^5_+: 0\le S(t)+ E(t) + R(t)+ D(t)\le \frac{\varLambda }{\mu },\, 0\le C(t)\le \left( \frac{\alpha \varLambda }{\mu \mu _0}\right) \right\} . \end{aligned}$$

For the well-posedness of the model, we have the following lemma.

#### Lemma 1

The set $\varGamma$ is positively invariant to system (1).

#### Proof

From the equation (3), we have

$$\begin{aligned} \frac{\text {d}N(t)}{\text {d}t}\le \varLambda -\mu N(t), \end{aligned}$$

and

$$\begin{aligned} \frac{\text {d}}{\text {d}t}\left( N(t)e^{\mu t}\right) \le \varLambda e^{\mu t}. \end{aligned}$$

Now integrating the above equation from $0$ to $t$, we have

$$\begin{aligned} N(t)\le N(0)e^{-\mu t}+\frac{\varLambda }{\mu }. \end{aligned}$$

Therefore, by using the theory of differential inequalities [18], we obtain $\lim \limits _{t\rightarrow \infty }\sup N(t)\le \frac{\varLambda }{\mu }$, i.e., the human population can not be more than $N(t)\rightarrow N(\infty )\le \frac{\varLambda }{\mu }$.

Further, from the last equation of model system (1) and using the fact, $R(t)\le \frac{\varLambda }{\mu }$ for large $t>0$, we obtain

$$\begin{aligned} \frac{\text {d}C(t)}{\text {d}t}+\mu _0 C(t)\le \left( \alpha \frac{\varLambda }{\mu }\right) . \end{aligned}$$

From the theory of differential inequality, we obtain

$$\begin{aligned} \lim \limits _{t\rightarrow \infty }\sup C(t)\le \left( \frac{\alpha \varLambda }{\mu \mu _0}\right) . \end{aligned}$$

This implies that $0\le C(t)\le \left( \frac{\alpha \varLambda }{\mu \mu _0}\right)$ for large $t\ge 0$. The proof is complete. $\square$

Hence, system (1) is considered mathematically and physically well posed in $\varGamma$ [25].

### Equilibria and their stability analysis

In this sub-section, we show the feasibility of all equilibria by setting the rate of change with respect to time *t* of all dynamical variables to zero. Model (1) has two feasible equilibria, which are listed as follows:

- The racism-free equilibrium (RFE) $E_1=\left( S^0,E^0,R^0,D^0,C^0\right) =\left( \frac{\varLambda }{C_0 \theta +\mu },0,0,\frac{\theta \varLambda C_0}{\mu ^2+\theta \mu C_0},C_0\right) .$
- The racism prevalence equilibrium (RPE) $E_2=\left( S^*,E^*,R^*,D^*,C^*\right)$. The existence of racism prevalence equilibrium point is computed after we have the threshold value ${\mathcal {R}}_0$.

#### Spreading threshold of the model

Here, we will find the basic reproduction number $({\mathcal {R}}_0)$ of model (1) using next-generation matrix approach [12]. We have the matrix of new racist ${\mathcal {F}}(X)$ and the matrix of transfer ${\mathcal {V}}(X)$. Let $X=\left( E,R,S,D,C\right) ^T,$ model (1) can be rewritten as:

$$\begin{aligned} \dfrac{dX}{\text {d}t}={\mathcal {F}}(X)-{\mathcal {V}}(X), \end{aligned}$$

where

$$\begin{aligned} {\mathcal {F}}(X)=\begin{pmatrix} \beta SR\\ 0\\ 0\\ 0\\ 0 \end{pmatrix},\, {\mathcal {V}}(X)=\begin{pmatrix} (\phi _d+\phi _r+\mu ) E\\ (\gamma +\mu ) R- \phi _r E\\ \beta SR+\theta SC+\mu S-\varLambda \\ \mu D-[\theta SC+ \gamma R+\phi _d E]\\ \mu _0(C-C_0)-\alpha R \end{pmatrix}. \end{aligned}$$

The Jacobian matrices of ${\mathcal {F}}(X)$ and ${\mathcal {V}}(X)$ at $E_1=\left( \frac{\varLambda }{C_0 \theta +\mu },0,0,\frac{\theta \varLambda C_0}{\mu ^2+\theta \mu C_0},C_0\right)$ are, respectively,

$$\begin{aligned} J{\mathcal {F}}(E_1)= \begin{pmatrix} F&{} 0 \\ 0 &{} 0 \end{pmatrix}, \,\, J{\mathcal {V}}(E_1)=\begin{pmatrix} V &{} 0 \\ J_1 &{} J_2 \end{pmatrix}, \end{aligned}$$

where

$$\begin{aligned} F=\begin{pmatrix} 0 &{} \frac{\beta \varLambda }{\mu +\theta C_0} \\ 0&{}0 \end{pmatrix}\,\text {and}\,V=\begin{pmatrix} \phi _d+\phi _r+\mu &{} 0 \\ -\phi _r &{}\gamma +\mu \end{pmatrix}. \end{aligned}$$

The inverse of *V* is computed as

$$\begin{aligned} {V}^{-1}\left( \begin{array}{cc} \frac{1}{\mu +\phi _d+\phi _r} &{} 0 \\ \frac{\phi _r}{(\gamma +\mu ) \left( \mu +\phi _d+\phi _r\right) } &{} \frac{1}{\gamma +\mu } \\ \end{array} \right) . \end{aligned}$$

The next-generation matrix ${\mathcal {K}}_L = F{V}^{-1}$ is given by

$$\begin{aligned} {\mathcal {K}}_L= \left( \begin{array}{cc} \frac{\beta \varLambda \phi _r}{(\gamma +\mu ) (C_0\theta +\mu ) \left( \mu +\phi _d+\phi _r\right) } &{} \frac{\beta \varLambda }{(\gamma +\mu ) (C_0 \theta +\mu )} \\ 0 &{} 0 \\ \end{array} \right) . \end{aligned}$$

Therefore, basic reproduction number is ${\mathcal {R}}_0 = \rho ({\mathcal {K}}_L) = {\text {max}}\left( |\mu |: \mu \in \rho ({\mathcal {K}}_L)\right)$ is spectral radius of matrix ${\mathcal {K}}_L$ and it obtained as follows:

$$\begin{aligned} {\mathcal {R}}_0= \frac{\beta \varLambda \phi _r}{(\gamma +\mu ) (\mu +\theta C_0) \left( \phi _d+\mu +\phi _r\right) }. \end{aligned}$$

#### Stability of the RFE

Here, we determine the result of linear stability of the equilibrium $E_1$ of model (1).

##### Theorem 2

If ${\mathcal {R}}_0 < 1$, the racism-free equilibrium $E_1$ of system (1) is locally asymptotically stable, and it is unstable if ${\mathcal {R}}_0 > 1$.

##### Theorem 3

If ${\mathcal {R}}_0\le 1$, then the racism-free equilibrium, $E_1$, of system (1) is globally asymptotically stable in $\varGamma$.

##### Proof

Let $W=(S,E,R,D,C)^T$, $\kappa =(\phi _d+\phi _r+\mu ),\, \xi =(\gamma +\mu )$, and consider a Lyapunov function:

$$\begin{aligned} {\mathcal {L}}(w)=\phi _r E+\kappa R. \end{aligned}$$

Differentiating ${\mathcal {L}}$ in the solutions of system (1), we get

$$\begin{aligned} \dot{{\mathcal {L}}}&=\phi _r {\dot{E}}+\kappa {\dot{R}},\\&=\phi _r\left( \beta S R- \kappa E\right) +\kappa \left( \phi _r E-\xi R\right) \\&=\left( \phi _r \beta S -\kappa \xi \right) R \\&=\kappa \xi \left( \dfrac{\phi _r \beta }{\kappa \xi } S -1\right) R. \end{aligned}$$

Therefore,

$$\begin{aligned} \dot{{\mathcal {L}}}&\le \kappa \xi \left( \dfrac{\phi _r \beta }{\kappa \xi } S^* -1\right) R=\kappa \xi \left( {\mathcal {R}}_0 -1\right) R,\,\,\text {since}\,\, S\le S(0)\, \text {and}\, S\in \varGamma . \end{aligned}$$

$\dot{{\mathcal {L}}}< 0$ whenever ${\mathcal {R}}_0 < 1$. Furthermore, $\dot{{\mathcal {L}}} = 0$ if and only if ${\mathcal {R}}_0 = 1$. Thus the largest invariant set in $\left\{ W\in \varGamma | \dot{{\mathcal {L}}}(E,R)=0\right\}$ is the singleton. By LaSalle’s Invariance Principle the racism-free equilibrium is globally asymptotically stable in $\varGamma$, completing the proof. $\square$

Theorem 3 completely determines the global dynamics of model (1) in when ${\mathcal {R}}_0\le 1$. It establishes the basic reproduction number ${\mathcal {R}}_0$ as a sharp threshold parameter. Namely, if ${\mathcal {R}}_0<1$, all solutions in the feasible region converge to the RFE $E_1$, and the racism will die out from the community irrespective of the initial conditions. If ${\mathcal {R}}_0>1$, $E_1$ is unstable and the system is uniformly persistent, and a racism spread will always exist.

#### Racism prevalence equilibrium stability

Here, we show the feasibility of racism prevalence equilibrium $E_2$. The values of $S^*, E^*, R^*, D^*$, and $C^*$ are obtained by solving the following set of algebraic equations:

4 $$\begin{aligned} \begin{aligned} \varLambda - \beta S^*R^* - \theta S^*C^*-\mu S^*=0,\\ \beta S^*R^*- (\phi _d+\phi _r+\mu ) E^*=0, \\ \phi _r E^*- (\gamma +\mu ) R^*=0, \\ \theta S^*C^*+ \gamma R^*+\phi _d E^* -\mu D^*=0,\\ \alpha R^*-\mu _0(C^*-C_0)=0. \end{aligned} \end{aligned}$$

After some algebraic calculations, we get the value of $E_2$ as:

$$\begin{aligned} S^*=&\dfrac{S^0}{ {\mathcal {R}}_0}, \qquad E^*=\dfrac{({\mathcal {R}}_0-1)\mu \mu _0 \xi }{\phi _r(\alpha \theta +\beta \mu _0)},\\ R^*=&\dfrac{({\mathcal {R}}_0-1)\mu \mu _0}{(\alpha \theta +\beta \mu _0)},\qquad C^*=\dfrac{({\mathcal {R}}_0-1)\alpha \mu }{(\alpha \theta +\beta \mu _0)},\\ D^*=&\dfrac{({\mathcal {R}}_0-1)(\beta \gamma \mu _0 \phi _r+\alpha \theta \kappa \xi +\beta \mu _0 \xi \phi _d)}{\beta \phi _r(\alpha \theta +\beta \mu _0)}. \end{aligned}$$

Therefore, there exists a unique positive solution only when ${\mathcal {R}}_0>1$. Thus, it has a unique racism prevalence equilibrium, $E_2$.

##### Theorem 4

If ${\mathcal {R}}_0 > 1$, then the racism prevalence equilibrium point $E_2$ of system (1) is locally asymptotically stable.

##### Proof

The Jacobian matrix of the model at $E_2$ is

5 $$\begin{aligned} J_*\left( E_2\right) =\left( \begin{array}{ccccc} -\mu {\mathcal {R}}_0 &{} 0 &{} -\dfrac{\beta S^0}{{\mathcal {R}}_0 } &{} 0 &{} -\dfrac{\theta S^0 }{{\mathcal {R}}_0 } \\ \dfrac{({\mathcal {R}}_0-1) \beta \mu \mu _0}{(\alpha \theta +\beta \mu _0)} &{} -\kappa &{} \dfrac{\beta S^0}{{\mathcal {R}}_0 } &{} 0 &{} 0 \\ 0 &{} \phi _r &{} -\xi &{} 0 &{} 0 \\ \dfrac{({\mathcal {R}}_0-1) \alpha \theta \mu }{(\alpha \theta +\beta \mu _0)} &{} \phi _d &{} \gamma &{} -\mu &{} \dfrac{\theta S^0 }{{\mathcal {R}}_0 } \\ 0 &{} 0 &{} \alpha &{} 0 &{}-\mu _0 \\ \end{array} \right) . \end{aligned}$$

From the Jacobian matrix (5), we get $\uplambda _1=-\mu$. Furthermore, the stability of the system could prove using the Routh-Hurwitz criteria. The characteristic polynomial of (5) is

6 $$\begin{aligned} P(\uplambda )=\uplambda ^4+a_1\uplambda ^3+a_2\uplambda ^2+a_3\uplambda +a_4=0, \end{aligned}$$

where

7 $$\begin{aligned} \begin{aligned} a_1&=\kappa +\mu {\mathcal {R}}_0 +\mu _0+\xi , \\ a_2&=\mu _0(\xi +\kappa )+{\mathcal {R}}_0\mu (\kappa +\mu _0+\xi ), \\ a_3&= \frac{\beta \mu \mu _0, ({\mathcal {R}}_0-1)}{(\beta \mu _0+\alpha \theta )}+\mu {\mathcal {R}}_0 \mu _0(\kappa + \xi ),\\ a_4&=\mu _0\mu \kappa \xi ({\mathcal {R}}_0-1). \end{aligned} \end{aligned}$$

The polynomial (6) has negative roots (eigenvalues) if all its coefficients terms are positive, or it satisfies Routh–Hurwitz criteria of stability [11]. From (7) we can verify that $a_1>0,\, a_4>0,\,a_1a_2-a_3>0$ and $a_3(a_1a_2-a_3)-a_1^2a_4>0$, when ${\mathcal {R}}_0>1$. Hence, according to the Routh–Hurwitz criterion, all roots have negative real parts. Thus, the racism equilibrium is asymptotically stable. The proof is complete. $\square$

The result tells as, if the initial values of any trajectory are near the equilibrium $E_2$, then the solution trajectories approach to the $E_2$ under the condition ${\mathcal {R}}_0>1$.

## Sensitivity analysis

We explore ${\mathcal {R}}_0$ sensitivity analysis of system (1) to determine the model robustness to parameter values. This is a strategy to identify the most significance parameters of the model dynamics. The normalized sensitivity index $\varUpsilon _\uplambda$ is given by:

$$\begin{aligned} \varUpsilon ^{{\mathcal {R}}_0}_{\uplambda }=\frac{\partial {\mathcal {R}}_0}{\partial \uplambda }\times \frac{\uplambda }{{\mathcal {R}}_0}. \end{aligned}$$

Thus normalized sensitivity indices for parameters are obtained as

8 $$\begin{aligned} \begin{aligned} \varUpsilon ^{{\mathcal {R}}_0}_{\beta }&=1,\qquad \varUpsilon ^{{\mathcal {R}}_0}_{\phi _r}=\dfrac{\phi _d+\mu }{\kappa },\qquad \varUpsilon ^{{\mathcal {R}}_0}_{\phi _d}=\dfrac{-\phi _d}{\kappa },\\ \varUpsilon ^{{\mathcal {R}}_0}_{\theta }&=\dfrac{-\theta C_0}{(\mu +\theta C_0)},\qquad \varUpsilon ^{{\mathcal {R}}_0}_{\gamma }=\dfrac{-\gamma }{\xi }. \end{aligned} \end{aligned}$$

From the sensitivity indices calculation results, we can identify some parameters that strongly influence the model dynamics. Parameters $\beta$ and $\phi _r$ have a positive influence on the basic reproduction number ${\mathcal {R}}_0$, that is, an increase in these parameters implies an increase in ${\mathcal {R}}_0$. While parameters $\phi _d,\,\theta ,$ and $\gamma$ have a negative influence on the basic reproduction number ${\mathcal {R}}_0$, that is, an increase in these parameters implies a decrease in ${\mathcal {R}}_0$.

Here, we illustrate graphically demonstration of model (1) parameters values sensitivity.

**Fig. 2:**
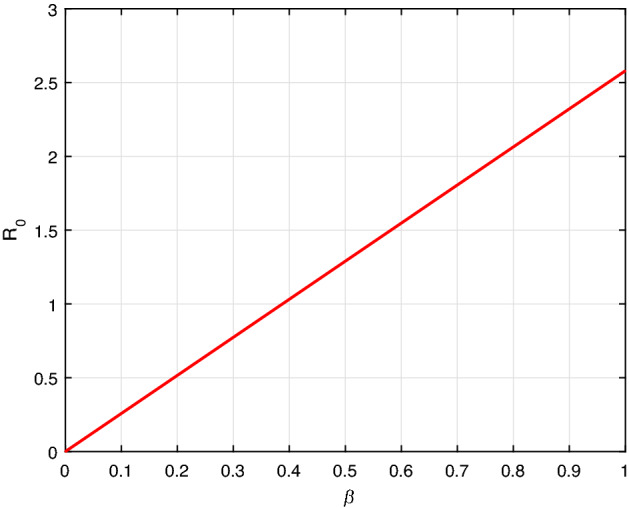
${\mathcal {R}}_0$ versus the parameter $\beta$

**Fig. 3:**
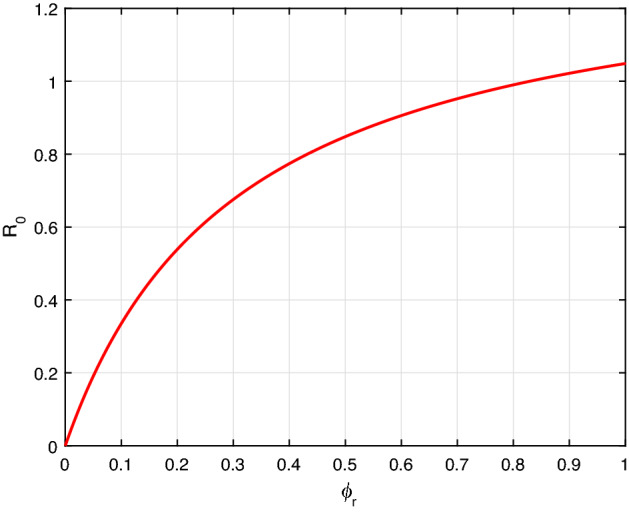
${\mathcal {R}}_0$ versus the parameter $\phi _r$

Figure 2 illustrates that the rise in contact rate $\beta$ facilitates the rapid spread of racism. Similarly, Fig. 3, despite racial extremeness rate $\phi _r$ also increase the propagation dynamics of racism. Further, applying an appropriate intervention measure reduces the contact and racial extremeness rate.

**Fig. 4:**
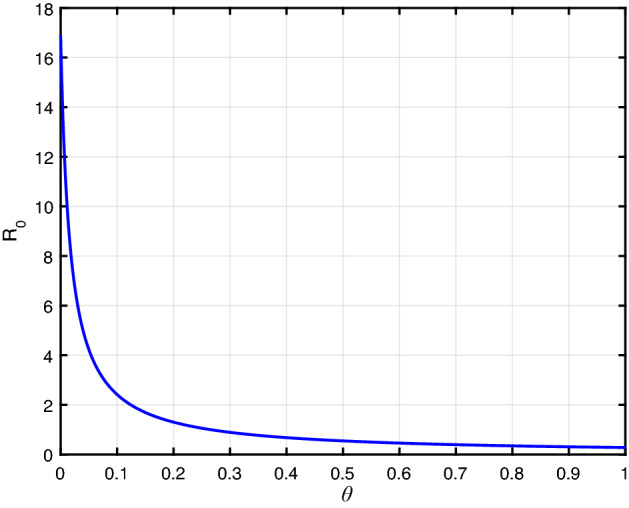
${\mathcal {R}}_0$ versus the parameter $\theta$

**Fig. 5:**
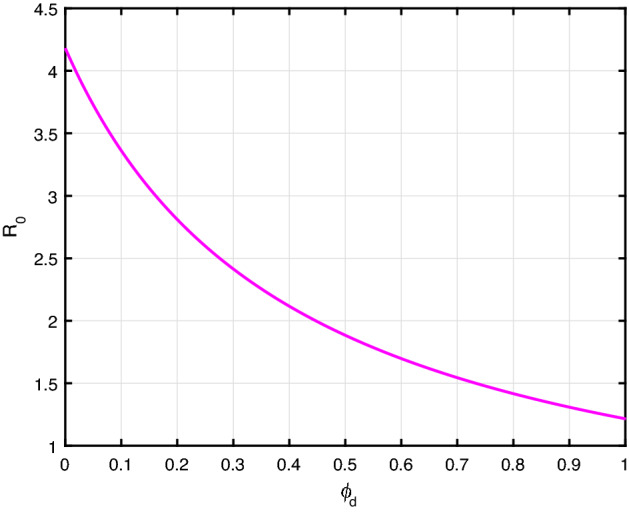
${\mathcal {R}}_0$ versus the parameter $\phi _d$

**Fig. 6:**
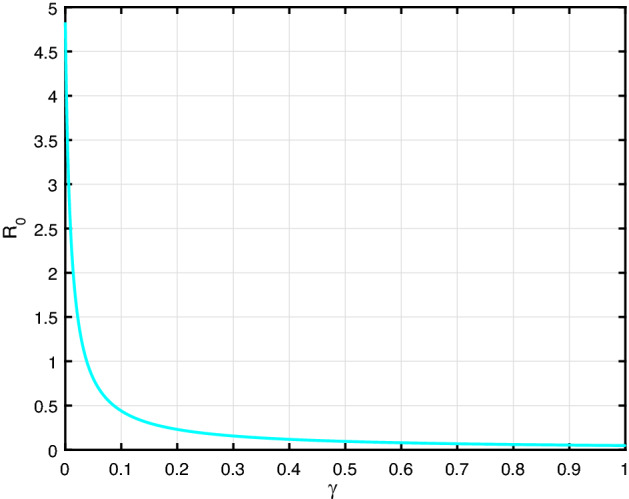
${\mathcal {R}}_0$ versus the parameter $\gamma$

Furthermore, the parameter $\theta$ is highly sensitive. The result provides community resilience is an effective control measure to eradicate the racism spread. From Figs. 4, 5, 6, we find that, if we increase the value of $\theta$, $\phi _d$, and $\gamma$, then racism spread decreases.

## Numerical simulations and analysis

In this section, we carry out numerical simulation of model (1) by using Matlab standard ordinary differential equations (ODEs) solver function ode45.

### General dynamics

Here, numerically illustrate the asymptotic behavior of model (1). Consider the initial conditions $S(0)=0.9,\,\, E(0)=0.06,\,\, R(0)=0.04,\,\, D(0)=0,\, \text {and}\, C(0)=0.1$.

**Fig. 7:**
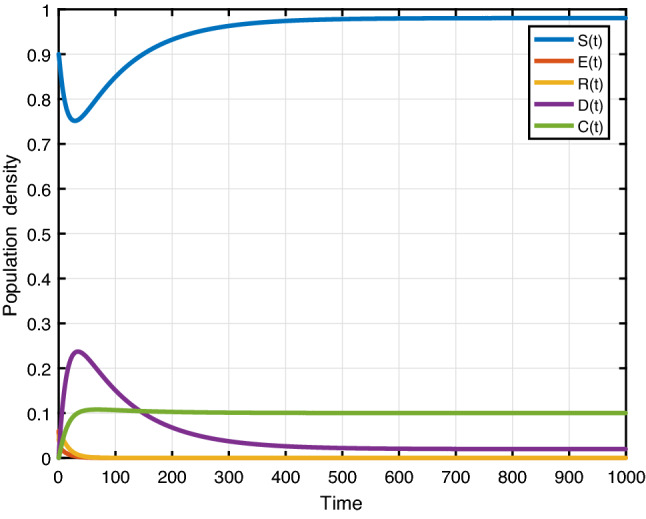
Evaluations over time (${\mathcal {R}}_0<1$)

**Fig. 8:**
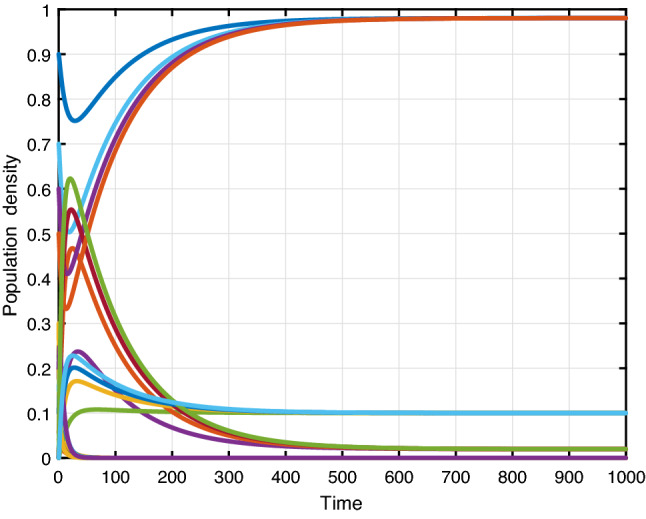
Varying initial values (RFE)

Figure 7 presents the trajectories of model (1), when $\beta =0.3,\, \phi _r=0.4,\,\phi _d=0.3,\,\gamma =0.2,\, \mu =0.01,\,\mu _0=0.01,\,\theta =0.01,\,\varLambda =0.01,\,\alpha =0.1,\, C_0=0.1$, thus the basic reproduction number ${\mathcal {R}}_0=0.789$. The trajectories converge to the racism-free equilibrium point $E_1$. Figure 8 shows the simulation of several solutions of system (1) with different randomly given initial values. Figure 8 shows that all of these solutions eventually converge to the racism-free equilibrium $E_1$. Furthermore, social stability is substantially continuous with the strengthening of community cohesion and unity through diversity. Moreover, the damages caused by the existence of racism will be removed. Finally, we have to get a racism-free community by eliminating racist activists from the environment.

**Fig. 9:**
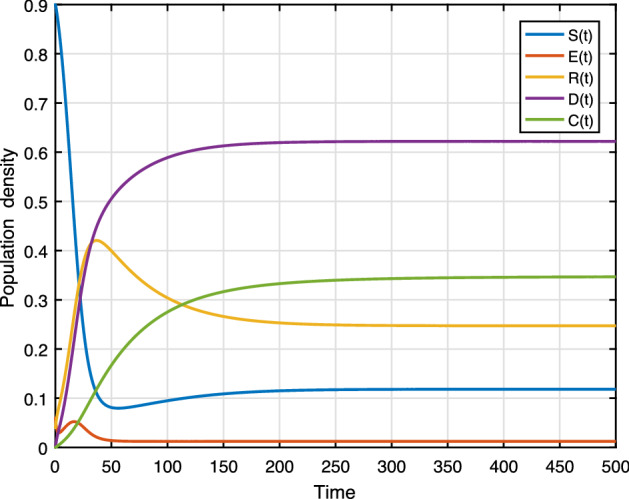
Evaluations over time (${\mathcal {R}}_0 >1$)

**Fig. 10:**
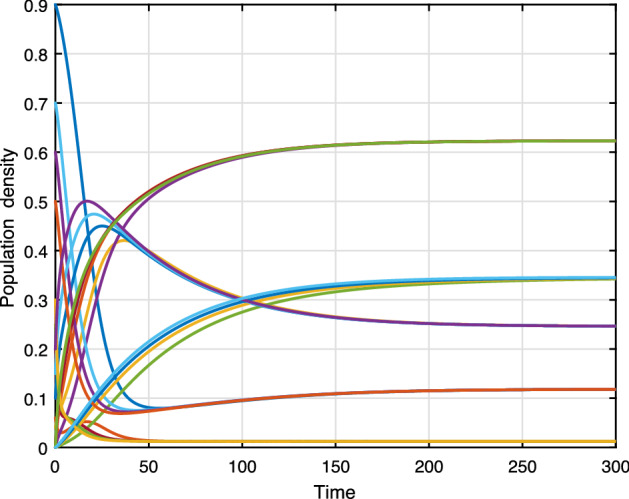
Varying initial values (RPE)

Figure 9 gives the trajectory simulation where $\beta =0.3,\, \phi _r=0.4,\, \phi _d=0.3,\,\gamma =0.01,\, \mu =0.01,\,\mu _0=0.01,\,\theta =0.01,\,\varLambda =0.01,\,\alpha =0.01, \,C_0=0.1$, the basic reproduction number is ${\mathcal {R}}_0=8.367$. We can see that even for a small fraction of the racist state at the beginning the racism persists in the community and stabilizes in time. This means that the trajectories converge to the racism prevalence equilibrium point. Figure 10 shows the simulation of its four solutions with varying initial values. The simulation has shown that all of these solution trajectories finally tend to the racism prevalence equilibrium $E_2$. Thus, as established in Theorem 4, the racism persists in the community whenever ${\mathcal {R}}_0>1$. Further, incompetent community resilience and less recovery of racist individuals lead to the prevalence of racism spread.

### Impact of effective contact rate

To evaluate the impact of effective contact in the system dynamics, we carry out some numerical simulations.

**Fig. 11:**
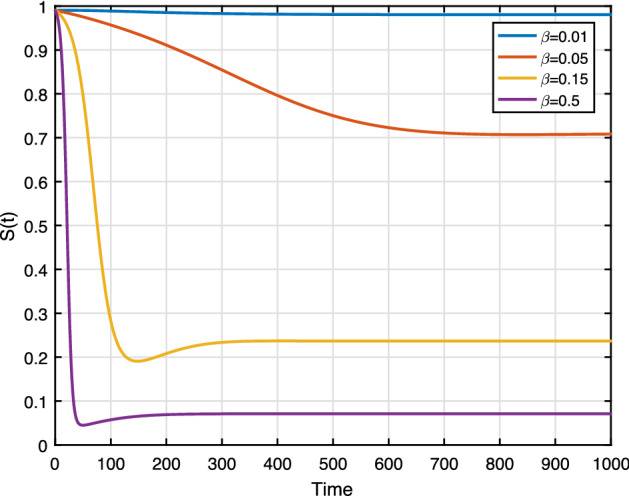
Effect of $\beta$ on *S*

**Fig. 12:**
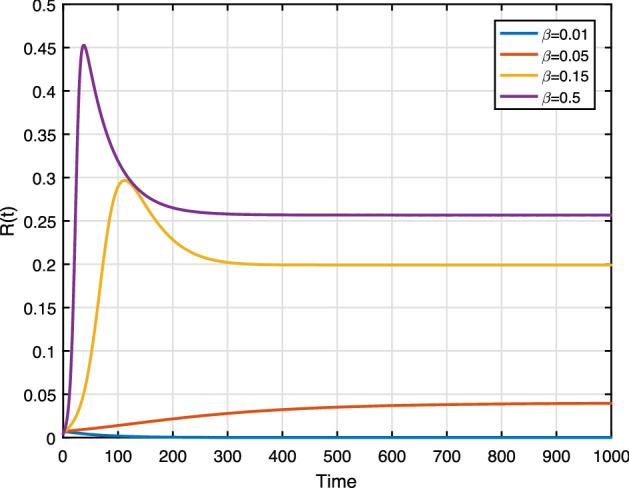
Effect of $\beta$ on *R*

We set the contact rate $\beta$ as 0.01, 0.05, 0.15 and $\beta =0.5$. From Fig. 12, we can see that racist individuals reach a higher peak level as $\beta$ increases. Figure 12 depicts the influence effective contact rate as shown in the sensitivity analysis. The result implying that containing contact between racist and susceptible individuals leads to reduce the racism spreading. Figure 11 shows the susceptible population also decreases whenever $\beta$ increases (almost $90\%$ of susceptible population will be racist where $\beta =0.5$).

### Impact of racial extremeness rate

Exposed individuals being racist with a rate of $\phi _r$, we conduct numerical simulations to shows the effect of $\phi _r$ on system (1).

**Fig. 13:**
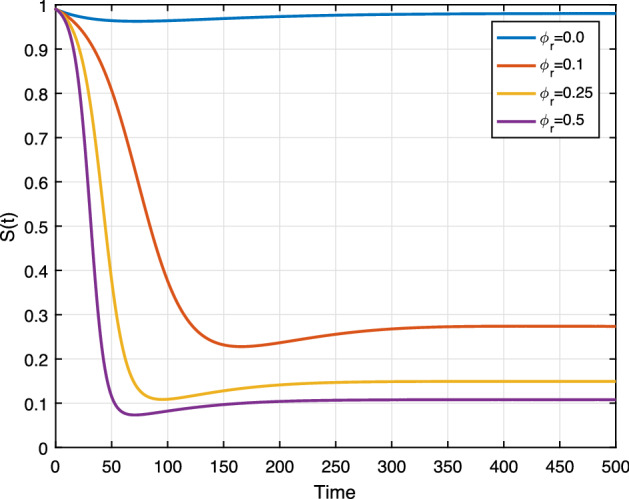
Effect of $\phi _r$ on *S*

**Fig. 14:**
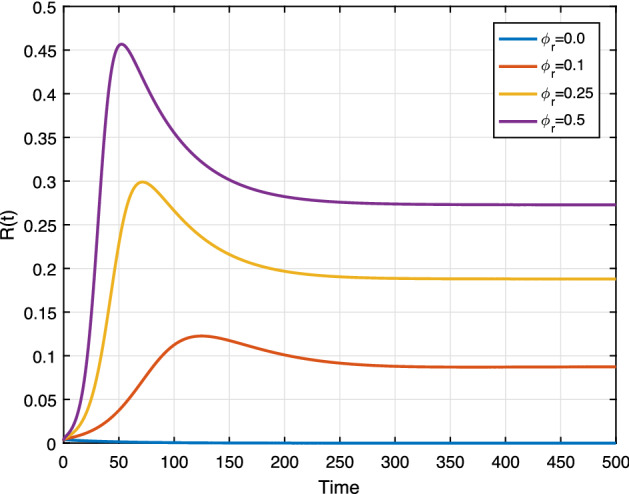
Effect of $\phi _r$ on *R*

We set the rate $\phi _r$ as 0.0, 0.1, 0.25 and 0.5. In Fig. 14, we can see that the racist individuals increase as $\phi _r$ increases. This figure shows the notable impact of $\phi _r$ as shown in Fig. 3, in the sensitivity analysis. The result implies that if the rate $\phi _r$ of hesitating state individual becomes large, then the racially racist individuals increase and the spread becomes persistent. Similarly, Fig. 13 provides the result susceptible individuals rapidly become the racist group whenever the rate $\phi _r$ increases.

### Impacts of community resilience

To evaluate the effects of community resilience, we carry out simulations by varying effect resilience campaign rate $\theta .$

**Fig. 15:**
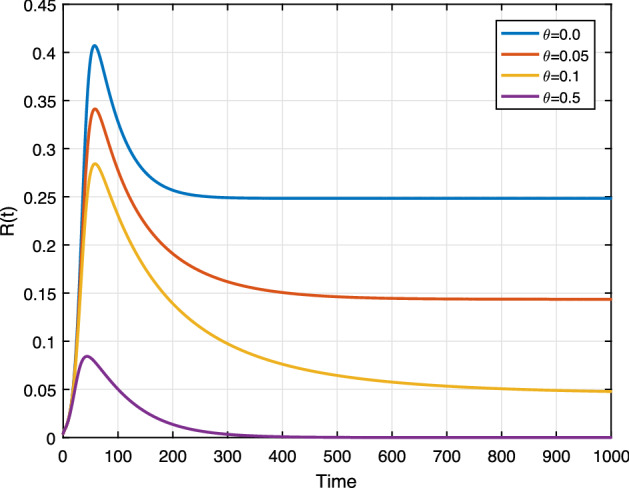
Effect of $\theta$ on *R*

Figure 15 shows the role of resilience campaign on the racist class. We set $\theta =0,0.01,0.05$ and $\theta =0.2$, it can observed that *R* solution trajectories converges to zero when $\theta$ values are increases. Thus, to intensify the coverage of community resilience, provide conquering the size of racist individuals. From the simulations, we learn efficient community resilience is a relevant control measure against racism propagation. This simulation result shows that more than $10\%$ effective resilience-building is required to conquer the propagation of racism.

## Discussion and conclusions

In this paper, we investigated the dynamics of racism spreading with control measures. A new *SERDC* racism spreading model with community resilience has been exhibited and simulated. We have studied the existence and stability of the equilibrium points of the offered model. Using the next-generation matrix approach, we have derived an explicit formula for the basic reproduction number, ${\mathcal {R}}_0$. Furthermore, we have shown that the RFE is globally asymptotically stable if ${\mathcal {R}}_0\le 1$ and unstable whenever ${\mathcal {R}}_0> 1$. For the case where ${\mathcal {R}}_0>1$, we have proven that there exists a unique racism prevalence equilibrium, which is locally asymptotically stable. The sensitivity analysis exhibits the significant control parameter of the model. The key parameters are the contact rate ($\beta$), the racial extremeness rate ($\phi _r$), recovery rate ($\gamma$), and effective community resilience campaign ($\theta$). Some numerical simulations were conducted to verify the theoretical analysis. Further, we demonstrate the impact of the model parameter value on the system dynamic. The general dynamics simulation result implies the racism spread is under control where ${\mathcal {R}}_0<1$, but persists in the population whenever ${\mathcal {R}}_0>1$.

Moreover, a resilient community would determine what it demands to conquer damage and then sustain to improve health, social, and economic systems. Similarly, multicultural communities are strengthening cohesion through diversity, and they enhance socio-economical benefits equally. Truly robust community resilience can promote the power of integrity and minimize damages by removing racism from the living environment.

## Data Availability

Not applicable.

## References

[1] Akdeniz, Y.: Stocktaking on efforts to combat racism on the internet. Background Paper. Intergovernmental Working Group on the Effective Implementation of the Durban Declaration and Programme of Action, Commission on Human Rights, E/CN **4**, 2006 (2006)

[2] Aldrich DP, Meyer MA (2015). Social capital and community resilience. Am. Behav. Sci.

[3] Barr S, Devine-Wright P (2012). Resilient communities: sustainabilities in transition. Local Environ..

[4] Bliuc AM, Faulkner N, Jakubowicz A, McGarty C (2018). Online networks of racial hate: a systematic review of 10 years of research on cyber-racism. Comput. Hum. Behav..

[5] Brauer F, Castillo-Chavez C, Castillo-Chavez C (2012). Mathematical models in population biology and epidemiology.

[6] Burnap P, Williams ML, Sloan L, Rana O, Housley W, Edwards A, Knight V, Procter R, Voss A (2014). Tweeting the terror: modelling the social media reaction to the woolwich terrorist attack. Soc. Netw. Anal. Mining.

[7] Burt CH, Simons RL, Gibbons FX (2012). Racial discrimination, ethnic-racial socialization, and crime: a micro-sociological model of risk and resilience. Am. Sociol. Rev..

[8] Chandra A, Acosta J, Meredith LS, Sanches K, Stern S, Uscher-Pines L, Williams M, Yeung D (2010). Understanding community resilience in the context of national health security.

[9] Cohen O, Goldberg A, Lahad M, Aharonson-Daniel L (2017). Building resilience: the relationship between information provided by municipal authorities during emergency situations and community resilience. Technol. Fore. Soc. Change.

[10] Dejen KM (2020). A model of coronavirus pandemic with public health intervention. Int. J. Math. Model. Comput..

[11] DeJesus EX, Kaufman C (1987). Routh-Hurwitz criterion in the examination of eigenvalues of a system of nonlinear ordinary differential equations. Phys. Rev. A.

[12] van den Driessche P (2017). Reproduction numbers of infectious disease models. Infect. Dis. Modelling.

[13] Hethcote HW (2000). The mathematics of infectious diseases. SIAM Rev..

[14] Houston JB (2015). Bouncing forward: assessing advances in community resilience assessment, intervention, and theory to guide future work. Am. Behav. Sci..

[15] Jakubowicz A, Dunn K, Mason G, Paradies Y, Bliuc AM, Bahfen N, Oboler A, Atie R, Connelly K (2017). Cyber racism and community resilience.

[16] Kermack WO, McKendrick AG (1927). A contribution to the mathematical theory of epidemics. Proc. R. Soc. London.

[17] Koya PR, Mamo DK (2015). Ebola epidemic disease: modelling, stability analysis, spread control technique, simulation study and data fitting. J. Multidiscipl. Eng. Sci. Technol..

[18] Lakshmikantham V, Leela S (1969). Differential and integral inequalities: theory and applications: volume I: ordinary differential equations.

[19] Magis K (2010). Community resilience: an indicator of social sustainability. Soc. Nat. Resour..

[20] Mamo DK (2020). Model the transmission dynamics of covid-19 propagation with public health intervention. Results Appl. Math..

[21] Mamo DK (2020). Modeling the spread dynamics of racism in cyberspace. J. Math. Model..

[22] Mason G, Czapski N (2017). Regulating cyber-racism. Melb. UL Rev..

[23] Pfefferbaum RL, Pfefferbaum B, Nitiéma P, Houston JB, Van Horn RL (2015). Assessing community resilience: an application of the expanded cart survey instrument with affiliated volunteer responders. Am. Behav. Sci..

[24] Sherrieb K, Norris FH, Galea S (2010). Measuring capacities for community resilience. Soc. Indicators Res..

[25] Yorke JA (1967). Invariance for ordinary differential equations. Math. Syst. Theory.

